# A Novel Low-Power and Soft Error Recovery 10T SRAM Cell

**DOI:** 10.3390/mi14040845

**Published:** 2023-04-13

**Authors:** Changjun Liu, Hongxia Liu, Jianye Yang

**Affiliations:** Key Laboratory for Wide Band Gap Semiconductor Materials and Devices of Education Ministry, School of Microelectronics, Xidian University, Xi’an 710071, China

**Keywords:** soft error, single-event upset (SEU), radiation hardness, low power

## Abstract

In SRAM cells, as the size of transistors and the distance between transistors decrease rapidly, the critical charge of the sensitive node decreases, making SRAM cells more susceptible to soft errors. If radiation particles hit the sensitive nodes of a standard 6T SRAM cell, the data stored in the cell are flipped, resulting in a single event upset. Therefore, this paper proposes a low-power SRAM cell, called PP10T, for soft error recovery. To verify the performance of PP10T, the proposed cell is simulated by the 22 nm FDSOI process, and compared with the standard 6T cell and several 10T SRAM cells, such as Quatro-10T, PS10T, NS10T, and RHBD10T. The simulation results show that all of the sensitive nodes of PP10T can recover their data, even when S0 and S1 nodes flip at the same time. PP10T is also immune to read interference, because the change of the ‘0’ storage node, directly accessed by the bit line during the read operation, does not affect other nodes. In addition, PP10T consumes very low-holding power due to the smaller leakage current of the circuit.

## 1. Introduction

With the growth of space exploration, the aerospace industry has expanded, introducing fields such as satellite communication, navigation, and space station construction. Processors are widely used in the control of aerospace electronic systems [1]. Multi-core processors have been designed to improve processing performance, but they require more cache [2]. SRAM, as the cache memory of the processor, plays an important role in the performance and area of the processor.

High-energy particles exist in space, and a single-event upset (SEU) caused by particle radiation is a major threat to the reliability of memory in nanometer CMOS technology [3,4]. When an energetic particle impacts the sensitive node of a semiconductor device, the electron-hole pair induced by the loss of energy will be separated by the depletion region of the reverse bias junction and collected by the sensitive node [5]. When the accumulated charge value is large enough and the time is long enough, it may cause the stored content to change [6]. In addition, as the minimum spacing between integrated circuit devices decreases rapidly, the flipping of a single particle may affect multiple nodes, resulting in the flipping of multiple nodes [6].

Researchers have used three structures of the same module to vote to solve the impact of SEU on memory. However, this method requires two additional redundant modules and a voting circuit, which increases the area and power loss [7]. Error correction code is a convenient solution to provide fault-tolerant protection in memory. However, due to the need for additional encoding and decoding circuits, error correction code will generate more power, area, and delay overhead [8]. In comparison, the use of SRAM-hardened cells imposes the lowest power consumption, area, and delay, so it is the most popular SEU solution.

Due to the advantages of the SRAM hardened unit scheme, researchers have designed various SRAM hardened units to solve the SEU problem. In 1996, Calin proposed the DICE cell, which is composed of 12 transistors and can resist full-type SEU inversion [9]. In 2009, Jahinuzzaman proposed the Quatro-10T cell, which can tolerate with 0→1 SEU of an SRAM cell [10]. In 2012, Jung proposed the PMOS-stacked 10T SRAM cell (PS10T) and the NMOS-stacked 10T SRAM cell (NS10T), which use stacked transistors to reduce circuit power consumption [11]. In 2018, Guo Jing proposed the radiation-hardened-by-design (RHBD) 10T SRAM cell, which can resist full-type SEU and partially resist charge-sharing effects [12]. In 2022, Pal proposed the Soft-Error-Aware Read-Stability-Enhanced Low-Power 12T (SARP12T) SRAM cell, which has low-power consumption in addition to full-type SEU resistance [13]. The goal of SRAM hardening is to improve the resistance to SEUs, decrease the area, and lower the power consumption.

However, the existing cells either provide only partial SEU immunity or require more transistor consumption. Moreover, the SRAM hardened unit requires multi-node disturbance recovery capability to cope with the more serious charge-sharing effect caused by CMOS technology.

This paper proposes a hardened SRAM cell, called PP10T, that can recover from soft errors. PP10T has full-type resistance to SEU, partial resistance to multi-node upsets caused by the charge-sharing effects, a small area, low-power consumption, read-disturbance-free and other advantageous characteristics. In Section 2, the function of PP10T is introduced and analyzed. In Section 3, PP10T is simulated and compared with other radiation-hardened cells. The conclusions of this paper are presented in Section 4.

## 2. Proposed PP10T Unit

**Figure 1:**
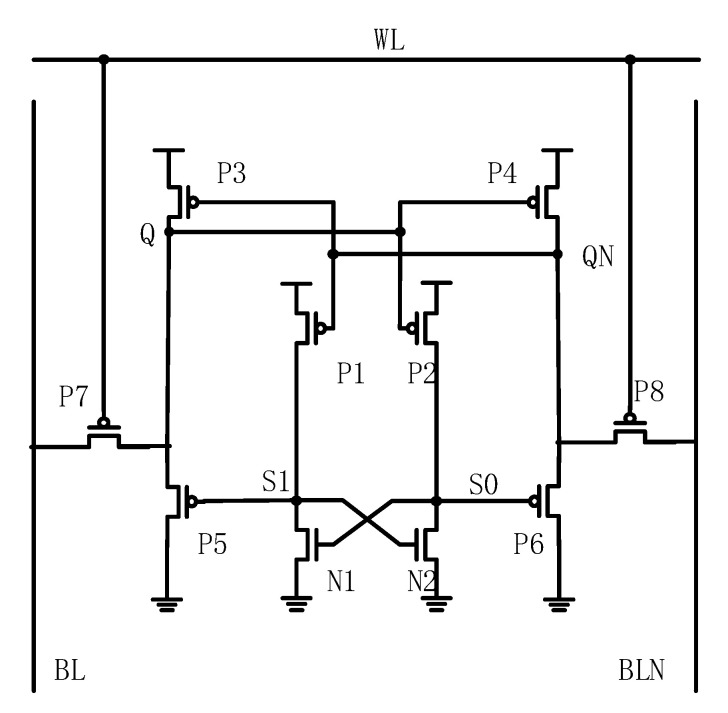
Schematic of the proposed PP10T SRAM cell.

This section introduces the structure of PP10T, which is shown in Figure 1. PP10T consists of 10 transistors, among which N1 and N2 are NMOS transistors, and P1 to P8 are PMOS transistors. To obtain excellent soft error recovery capability, the size of the pull-up transistor is larger than that of the pull-down transistor. Nodes Q, QN, S0, and S1 are data storage nodes, nodes Q and QN store the opposite data, and nodes S0 and S1 also store the opposite data.

### 2.1. Reading and Writing of PP10T

All of the analyses in this section take Q = S1 = “1”, QN = S0 = “0” as the initial state for illustration. Because the circuit is symmetrical, when storing the opposite data, the analysis is similar.

During the read operation, PP10T transmits the logical values of storage nodes Q and QN to the external port through bit lines, BL and BLN. Before the read operation, the BL and BLN are pre-charged to the logic “1”. During the read operation, the word line, WL, changes from “1” to “0”, the access transistors P7 and P8 are turned on, and the nodes Q, QN, S0, and S1 maintain their previous logical values. In this process, BLN starts to discharge under the discharge path formed by the conduction of P8 and P6. The off P5 will not make P7 and P5 form a conductive path, so the logic value of BL is still “1”. The differential sensitive amplifier identifies and amplifies the potential difference between BL and BLN, finally outputting the high and low levels to complete the reading operation.

During the writing operation, BL is set to “0”, BLN is set to “1”, and WL is set to “0”. The transistors P7 and P3 are turned on, and the size of the transistor P7 is larger than P3, so the voltage of node Q begins to change to “0”. The decrease of Q potential makes P2 and P4 open, and then P2 and P4 are charged. Because the size of the pull-up transistor is larger than that of the pull-down transistor, the potential of S0 and QN increases. The increase in the potential of S0 makes P6 close, and the value of QN becomes “1”. The change in the value of QN makes P1 and P3 close, and because the potential of S0 increases, N1 opens, which makes S1 become “0”. After S1 becomes “0”, P5 opens, and N2 closes. As P2 and P5 are conducted, S0 becomes “1”, and Q becomes logic “0”. At this time, all of the storage nodes complete the corresponding changes, and data “0” is successfully written to the SRAM cell.

During the holding operation, WL is “1”, and access transistors P7 and P8 are off. Transistor P3 is turned on, pulling node Q up to keep the logic value of node Q at “1”; transistor P1 is turned on, pulling node S1 up to keep the logic value of node S1 at “1”; transistor P6 is turned on, pulling node QN down to keep the logic value of node QN at “0”; transistor N2 is turned on, pulling node S0 down to keep the logic value of node S0 at “0”; and other transistors are turned off. The SRAM cell maintains the current storage state through the feedback mechanism composed of nodes Q, QN, S0 and S1.

### 2.2. SEE Analysis of PP10T 

This section takes Q = S1 = “1”, QN = S0 = “0” as an example to analyze the behavior of SEU after radiation particles hit storage nodes Q, QN, S0, and S1.

When a radiation particle strikes the node Q and the PMOS transistor, only a positive transient pulse is generated [14]. Only one “1→1” transient pulse can be generated at node Q, which does not change the logical value of node Q, so this node is not sensitive. When an SEU occurs at node QN, node QN changes from “0” to “1”, and transistors P3 and P1 are temporarily turned off. However, this does not affect the state of other transistors and storage nodes. With the help of transistor P6, the node QN is recovered. When an SEU occurs at node S1, the state of S1 changes from “1” to “0”. At this time, transistor N2 is temporarily turned off and transistor P5 is turned on. Transistors P5 and P3 are turned on at the same time. Because P3 is larger than P5, the logic value of node Q remains unchanged at “1”, and the node S0 remains unchanged at “0”. Finally, S1 is recovered. When an SEU occurs at node S0, node S0 changes from “0” to “1”, which turns on transistor N1 and turns off transistor P6. At this time, transistors P1 and N1 are turned on. Since P1 is wider than N1, the node S1 remains unchanged. When P6 is turned off, QN does not change due to the capacitive effect, and finally, the value of S0 is restored by the pull-down transistor N2. When nodes S0 and S1 change, the change of node S0 shuts down P6, but this does not affect other nodes. When S1 overturns, P5 opens. Since P3 is larger than P5, the logic value of node Q does not change. Finally, the node S1 is recovered by pull-up transistor P1, and then S0 is recovered by pull-down transistor N2.

Because this circuit is symmetrical, when the initial storage of node QN is “0”, the analysis will obtain similar results.

## 3. Analysis of Simulation Results

### 3.1. Verification of PP10T Read/Write Function

**Figure 2:**
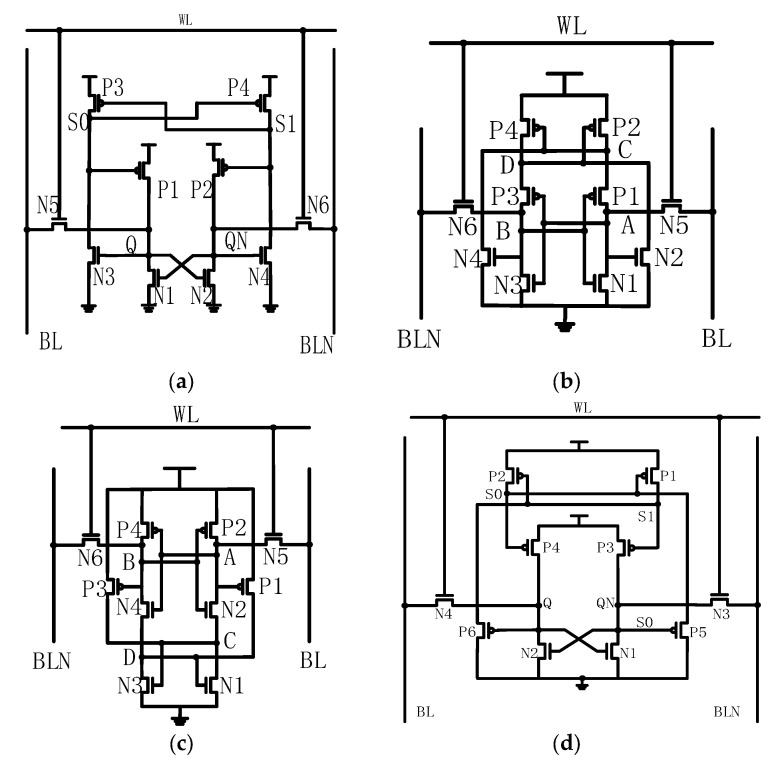
Hardened SRAM cells: (**a**) Quatro-10T; (**b**) PS10T; (**c**) NS10T; (**d**) RHBD10T.

In this paper, the Global Foundries 22 nm FDSOI process is used to simulate PP10T through the Hspice tool. This paper also simulates the standard 6T unit and the Quatro-10T, PS10T, NS10T, and RHBD10T cells. Under the condition of ensuring correct functions, transistors adopt similar sizes. The structure of each contrast unit is shown in Figure 2. The Quatro-10T memory cell uses negative feedback to prevent SEU flips of 1→0. PS10T and NS10T reduce the leakage current while preventing SEU by adding stacked transistors. The RHBD10T SRAM cell has complete single-event, single-node upset immunity.

**Figure 3:**
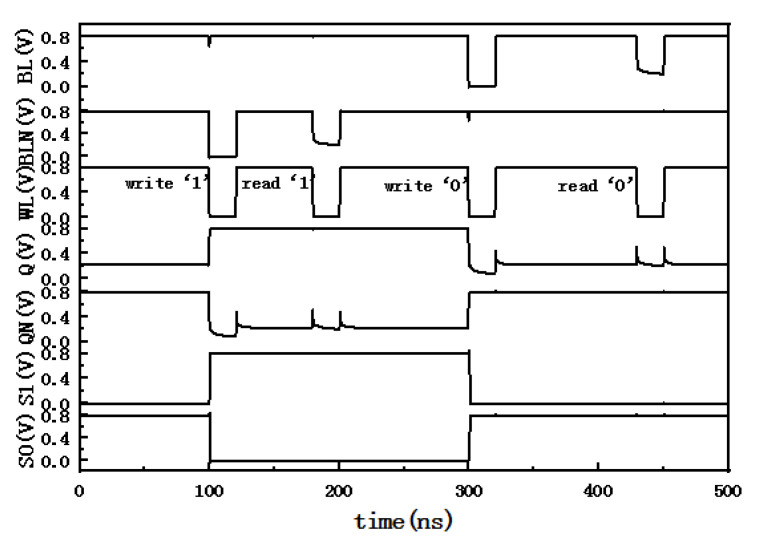
Write and read operation of PP10T.

PP10T read/write simulation is shown in Figure 3. Write operations are performed at 100 ns and 300 ns respectively, and read operations are performed at 180 ns and 430 ns, respectively. The read operation causes interference to the logic “0” potential node, but because the gate of the two PMOS devices is connected to the logic “0” node, the voltage rise of the node can only turn off the two PMOS devices at worst, without changing the storage state of the other nodes.

### 3.2. Write/Read Access Time Comparison

**Figure 4:**
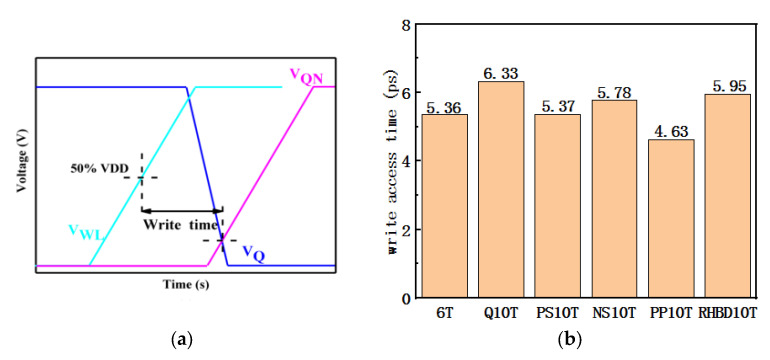
Write access time. (**a**) Measuring method. (**b**) Write access time comparison.

The write access time is characterized by the time difference between the time when WL reaches VDD/2 and the time when Q and QN intersect [15], as shown in Figure 4a. The write access time depends on the size ratio of the access transistor to the pull-up transistor. The larger the access transistor, the faster the write time. Figure 4b shows that the write time of the proposed PP10T cell is 86.4%, 73.1%, 86.2%, 80.1%, and 77.8% that of 6T, Quatro-10T, PS10T, NS10T, and RHBD10T, respectively. Among the comparison cells used in this paper, because the ratio of the access transistor to the pull-up transistor of PP10T is the largest and the size of the access transistor is also the largest, the corresponding write delay time is the smallest.

**Figure 5:**
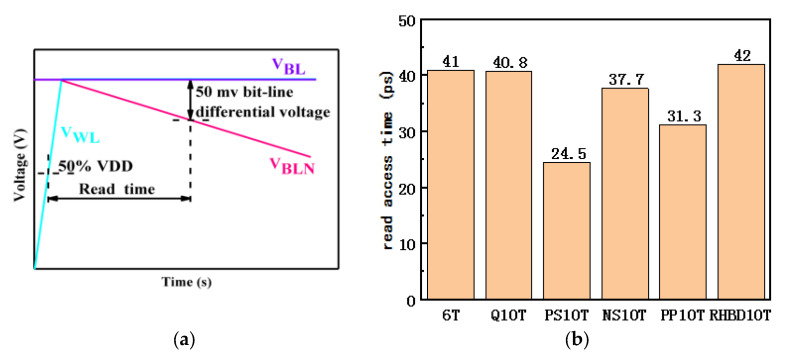
Read access time. (**a**) Measuring method. (**b**) Read access time comparison.

The measurement method of read access time is to calculate the time difference between the time when WL reaches VDD/2, and the time when the voltage difference between BL and BLN reaches 50 mv [15], as shown in Figure 5a. The read access time is strongly dependent on the read current through the access and pull-down transistors. From Figure 5b, it can be found that the reading time of the PP10T cell proposed in this paper is small, which is 76.3%, 76.7%, 127.8%, 83%, and 74.5% that of 6T, Quatro-10T, PS10T, NS10T, and RHBD10T, respectively. To obtain an excellent soft error recovery capability, PP10T uses a PMOS transistor as a pull-down transistor. Although the conductivity of PMOS is weaker than that of NMOS, it reduces the read access time of the storage unit due to the larger size of the access transistor.

### 3.3. Static Noise Margin Comparison

**Figure 6:**
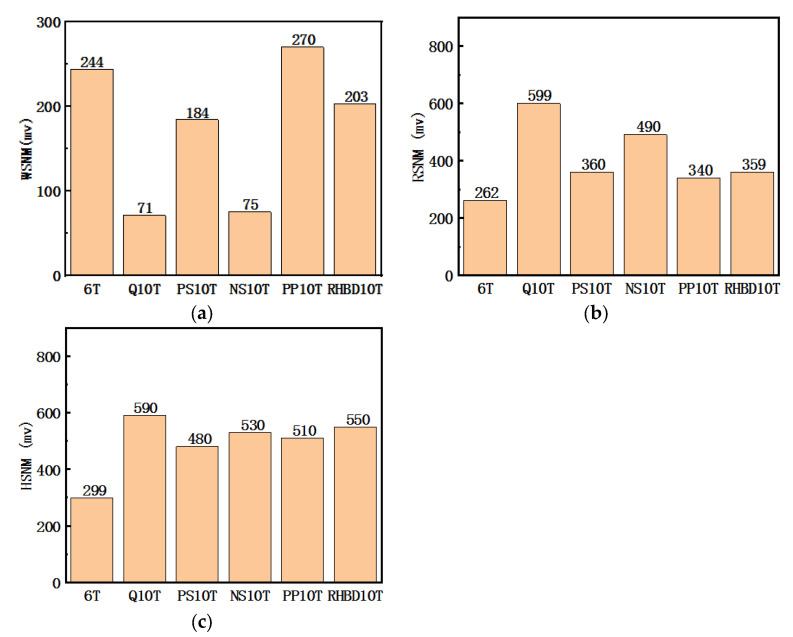
Static noise margin comparison: (**a**) WSNM, (**b**) RSNM., and (**c**) HSNM.

The write static noise margin (WSNM) is characterized by the voltage difference between WL and VDD when Q and QN intersect [16]. Because PP10T uses PMOS as the read transistor, the corresponding write static noise margin is the voltage difference between WL and GND when Q and QN intersect. According to the definition of write access time, a cell with a shorter write access time requires less time to alter its stored data, so a lower voltage occurs at WL. Therefore, a cell with a shorter write access time also has a larger WSNM. WSNM has the opposite law with the value of write access time. The result of each cell is shown in Figure 6a. PP10T has the largest write static noise tolerance, which is 110.6%, 380.3%, 146.7%, 360%, and 133% that of 6T, Quatro-10T, PS10T, NS10T, and RHBD10T, respectively.

Read stability is characterized by the read static noise margin (RSNM). The read static noise margin is the maximum DC noise voltage that SRAM can withstand during the read operation. Figure 6b shows that the read static noise margin of the PP10T cell is 129.7%, 56.7%, 94.4%, 69.4%, and 94.7% that of 6T, Quatro-10T, PS10T, NS10T, and RHBD10T, respectively. During the read operation, the rising voltage generated on the “0” storage node may cause the storage state of the SRAM cell to change. The higher the voltage rise generated on the ‘0’storage node, the higher the probability that the unit suffers from read interference. Since the proposed unit uses a PMOS transistor to drive the “0” node, there is an initial PMOS device voltage threshold at the “0” node, so its RSNM is smaller than other units. The RSNM value of PP10T is larger than that of 6TSRAM, because its CR value is larger than that of the 6T cell.

The stability in the hold state is characterized by the hold static noise margin. Figure 6c shows the hold static noise margin of each cell, in which the proposed PP10T has the highest static noise tolerance. The hold static noise margin of PP10T is 170.6%, 86.4%, 106.3%, 96.2%, and 92.7% that of 6T, Quatro-10T, PS10T, NS10T, and RHBD10T, respectively. In the holding state, the stronger the driving ability of the storage node, the lower the susceptibility to interference. Since the drive of the storage node “0” is slightly weaker, the retention tolerance of the proposed unit is lower than that of some of the other cells. However, due to the existence of the feedback loop, the HSNM of the PP10T unit is still higher than that of the 6T and PS10T cells.

### 3.4. Hold Power Comparison

**Figure 7:**
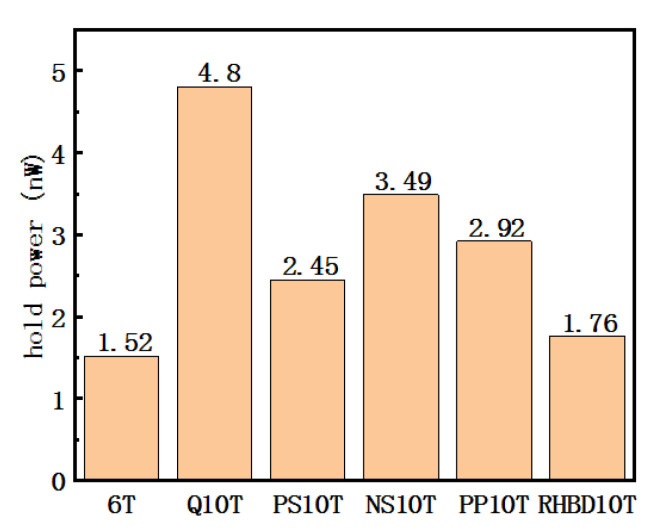
Hold power consumption comparison.

Hold power consumption accounts for most of the total power consumption of the SRAM cell, which is mainly caused by the leakage current in the storage structure and bit lines. The comparison of hold power consumption of all of the considered cells is shown in Figure 7. The hold power consumption of PP10T is 192.1%, 60.8%, 119.2%, 83.7%, and 165.9% that of 6T, Quatro-10T, PS10T, NS10T, and RHBD10T, respectively. Maintaining power consumption is positively correlated with leakage current and supply voltage. The magnitude of the current is related to the size of the device and the structure of the circuit. Quatro-10T has the highest power consumption because it has the most leakage paths and a relatively high current on the path. NS10T and PS10 reduce power consumption due to fewer leakage paths and stacked transistors. Because PP10T and RHBD use PMOS as a stable structure, and the electron mobility of PMOS devices is lower than that of NMOS devices, they have lower leakage currents and lower leakage power consumption.

### 3.5. SEU Recovery Verification

The model based on the Weibull function can capture various transient waveforms in the circuit, so it is used as a fault injection model for circuit-level SEE [17]. In this paper, the expression of the Weibull function model is:

(1) $$I\left(t\right)=\;a\cdot \left(\frac{c-1}{c}\right){\;}^{\left(\frac{1-c}{c}\right)\;}\cdot \left(\frac{t}{b}\right){\;}^{c-1}\cdot e^{-\left(\left(\frac{t}{b}\right){\;}^{c}-\frac{c-1}{c}\right)\;}\;,$$

where t is the time variable, a, b, and c are the shape parameters of the Weibull function, and the solution expression is:

(2) $$\left\{\begin{array}{l} a=H\\ b=\left(\frac{A\cdot c}{a}\right)\cdot {\left(\frac{c-1}{c}\right)}^{\frac{c-1}{c}}\cdot e^{\left(\frac{1-c}{c}\right)}\\ c=\frac{1}{1+\ln\left(1-P\right)}\\ P=\frac{AP}{A} \end{array}\right.$$

In this formula, *AP* represents the integration of the actual pulse current from 0 to tp, tp represents the time corresponding to the peak of the actual pulse current, *A* represents the integration of the actual current pulse and time, and *H* represents the peak of the actual pulse current.

**Figure 8:**
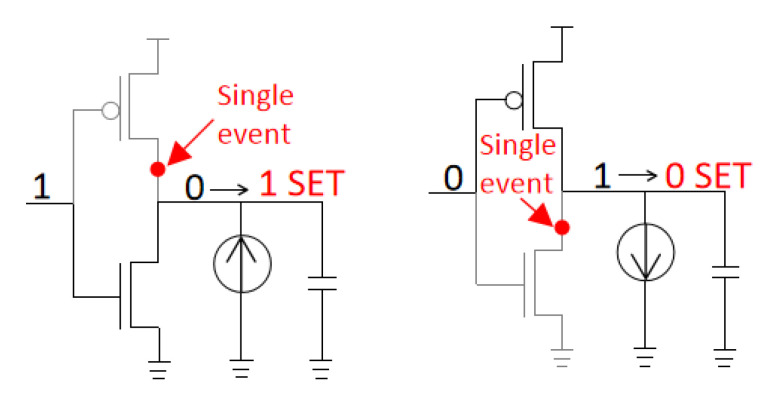
Circuit simulation fault injection.

Compared with the traditional double exponential current source, the Weibull current source has a higher fitting accuracy and is closer to the real pulse current curve generated after a heavy ion incident. Therefore, this current source is selected for simulation. Figure 8**.** shows the circuit-level fault injection method, which simulates the SEU phenomenon by adding the current source to the circuit-sensitive node.

**Figure 9:**
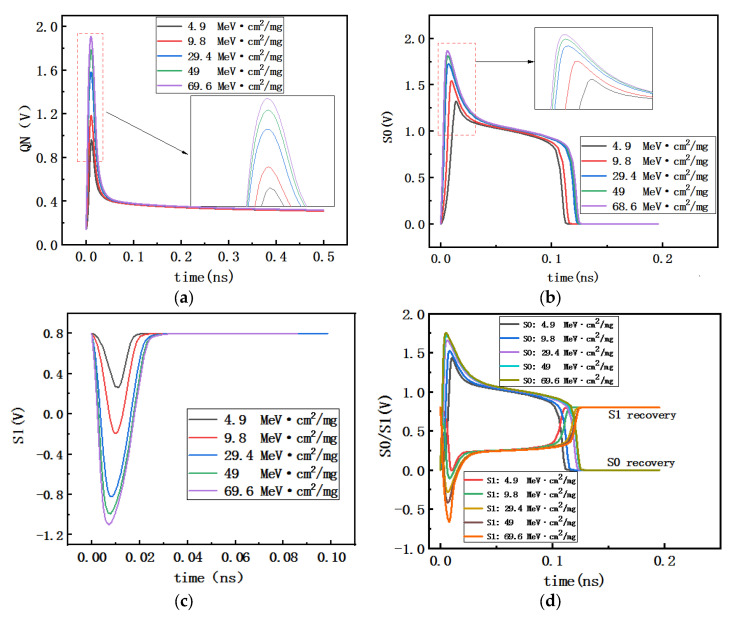
SEE analysis. (**a**) SEU at node QN.; (**b**) SEU at node S0; (**c**) SEU at node S1; (**d**) SEUs at node S0S1.

When heavy ions with different energies are incident at each sensitive node of the proposed cell, the behavior of soft error recovery is shown in Figure 9. Figure 9a–c shows the recovery of the three sensitive nodes, QN, S0, and S1, when encountering the single-event effect when the stored data is 1. Figure 9d shows the recovery of S0 and S1 caused by the charge-sharing effect when soft errors occur simultaneously. It can be seen from Figure 9. that the PP10T proposed can recover the SEU of a single node, and it also somewhat immunizes against the charge-sharing effect.

Table 1 presents a comparison between the reinforcement capacity of PP10T and other cells. When the incident ion LET reaches 1.96 MeV·cm^2^/mg, the storage state of the 6T unit will flips. This result is similar to the result of reference [18]. The difference between the results arises because the size of the two 6T SRAM cells is not exactly the same. The PS-10T and Quatro-10T only support the recovery from 1→0 SEU, and NS-10T only supports the recovery from 0→1 SEU. RHBD10T and PP10T can recover 1→0 and 0→1 inversions, and they can still recover when SEUs occur simultaneously at nodes S0 and S1. The simulation results also show that when the incident ion LET reaches 69 MeV·cm^2^/mg, RHBD10T will flip, but PP10T can still be restored to the correct state.

## 4. Conclusions

In this paper, a new 10T SRAM cell based on the 22 nm FDSOI process is proposed. Compared with the previous 10T hardened storage unit, the proposed unit can recover soft errors that occur in any sensitive node. The penalty introduced by the proposed 10T unit is to increase the area of the access transistor, using a PMOS transistor to transmit logic 0, which may affect some applications with strict area limits and full swing requirements of the bit line voltage. The simulation results show that the proposed PP10T unit provides a good balance between performance, area, power, and reliability, and it is a good choice for anti-radiation applications. 

The problem with the proposed unit is that it uses PMOS to drive the logic “0”, causing the line voltage change not to be in full swing. This means that the identification range of the sensitive amplifier of the peripheral circuit is not as large as that of the conventional unit. In addition, the reliability, power, access time, and static noise margin of the SRAM hardened unit conflict with the requirements for transistor size. Therefore, designers should choose the transistor size that provides the best trade-off between design goals and other performance metrics.

## Figures and Tables

**Table 1:** 10T hardened cell comparison.

CELL	6T	Quatro-10T	PS10T	NS10T	PP10T	RHBD10T
SEU (1→0)	No	Yes	Yes	No	Yes	Yes
SEU (0→1)	No	No	No	Yes	Yes	Yes
SEMU	No	No	No	No	Yes	Yes
LET (MeV cm2/mg)	1.96	>69	>69	>69	>69	<69

## Data Availability

Not applicable.
